# Perspectives of Frailty and Frailty Screening: Protocol for a Collaborative Knowledge Translation Approach and Qualitative Study of Stakeholder Understandings and Experiences

**DOI:** 10.1186/s12877-017-0483-7

**Published:** 2017-04-17

**Authors:** Mandy M. Archibald, Rachel Ambagtsheer, Justin Beilby, Mellick J. Chehade, Tiffany K. Gill, Renuka Visvanathan, Alison L. Kitson

**Affiliations:** 1 Adelaide Nursing School, Faculty of Health and Medical Sciences, The University of Adelaide, Adelaide, SA 5005 Australia; 2 Torrens University, Level 1, 220 Victoria Square, Adelaide, SA 5000 Australia; 3 Discipline of Orthopaedics & Trauma, Royal Adelaide Hospital, The University of Adelaide, Level 4 Bice Building, Adelaide, SA 5005 Australia; 4 Adelaide Medical School, South Australian Health and Medical Research Institute, Level 7, North Tce, Adelaide, SA 5000 Australia; 5 Floor 8B, The Queen Elizabeth Hospital, 28 Woodville Road, Woodville, SA 5011 Australia; 6 National Health and Medical Research Council Centre of Research Excellence in Trans-Disciplinary Frailty Research to Achieve Healthy Ageing, Adelaide, Australia

**Keywords:** Frailty, Ageing, Qualitative, Arts-based, Knowledge translation, Screening, Focus groups

## Abstract

**Background:**

Accompanying the unprecedented growth in the older adult population worldwide is an increase in the prevalence of frailty, an age-related clinical state of increased vulnerability to stressor events. This increased vulnerability results in lower social engagement and quality of life, increased dependency, and higher rates of morbidity, health service utilization and mortality. Early identification of frailty is necessary to guide implementation of interventions to prevent associated functional decline. Consensus is lacking on how to clinically recognize and manage frailty. It is unknown how healthcare providers and healthcare consumers understand and perceive frailty, whether or not they regard frailty as a public health concern; and information on the indirect and direct experiences of consumer and healthcare provider groups towards frailty are markedly limited.

**Methods:**

We will conduct a qualitative study of consumer, practice nurse, general practitioner, emergency department physician, and orthopedic surgeons’ perspectives of frailty and frailty screening in metropolitan and non-metropolitan South Australia. We will use tailored combinations of semi-structured interviews and arts-based data collection methods depending on each stakeholder group, followed by inductive and iterative analysis of data using qualitative description.

**Discussion:**

Using stakeholder driven approaches to understanding and addressing frailty and frailty screening in context is critical as the prevalence and burden of frailty is likely to increase worldwide. We will use the findings from the *Perceptions of Frailty and Frailty Screening* study to inform a context-driven identification, implementation and evaluation of a frailty-screening tool; drive awareness, knowledge, and skills development strategies across stakeholder groups; and guide future efforts to embed emerging knowledge about frailty and its management across diverse South Australian contexts using a collaborative knowledge translation approach. Study findings will help achieve a coordinated frailty and healthy ageing strategy with relevance to other jurisdictions in Australia and abroad, and application of the stakeholder driven approach will help illuminate how its applicability to other jurisdictions.

## Background

Frailty in older people is a significant challenge facing health systems today. Broadly recognized as an age-related clinical state of increased vulnerability to stressor events, the international prevalence of frailty ranges from 4.9% to 27.3%, with pre-frailty prevalence ranging between 34.6% and 50.9% [1]. In Australia, it is estimated that by 2050, four million Australians aged 70 years and older will either be frail or at-risk of frailty [2]. In South Australia, the prevalence of frailty is likely to exceed state averages, given that it has the oldest population of all the Australian states [3].

The impact of frailty on individuals, families, and health systems is far reaching. Frail individuals are more likely to have significant disability, morbidity and dependence, experience social isolation and an eroding self-confidence, and to develop a ‘frailty-identity’ [1, 4, 5]. Loss of independence in community settings often translates into increased caregiving responsibilities for family members, contributing to caregiver burnout, loss of income, and familial stress– factors that can be compounded by changes in the structure and function of contemporary families [6]. Governments and health systems are strained by the increased long-term care needs, medical costs, and social expenditures for frail older adults [7, 8]. Multi-level approaches to preventing, identifying, and managing frailty are therefore critical to reducing frailty’s multidimensional impact.

Identifying pre-frail and frail older adults is not only feasible but is also a necessary precursor of delivering effective interventions designed to halt and in some cases, reverse, functional decline [9–12]. Evidence is mounting on the importance of identifying frailty early using appropriate screening tools [13]. Despite this, no frailty-screening tool has been routinely implemented into clinical practice in Australia. Preceding this challenge is a lack of consensus on how frailty should be addressed in clinical environments [14]. Data on what constitutes “appropriateness” in each geographic and practice context is lacking, as is agreement on when screening should be conducted and by whom (e.g., practice nurses, general practitioners, older adults using self-assessment).

As such, a second critical component to improving the management of frailty is generating a broad understanding of how key stakeholders, including the public and healthcare providers, perceive frailty, frailty screening, and associated prevention and management interventions. It is unknown whether the public and healthcare provider groups are attuned to frailty as a public health concern and understandings of their direct and indirect experiences of frailty are markedly limited, particularly in the Australian setting [2, 15]. Few studies have explored how frail and well older adults understand and experience frailty [4, 5], and there literature is lacking on healthcare providers understandings. As such, it is unclear whether healthcare providers’ understanding of frailty aligns with the experiences of well and frail older adults. Understanding these diverse perspectives is an integral step to rectifying potential misalignments and improving the appropriateness and effectiveness of health service provision for older adults along the frailty spectrum.

In this paper we describe the protocol of a transdisciplinary study designed to understand how older adults and various healthcare provider groups perceive frailty and frailty screening in South Australia. Although the evidence base for public and healthcare providers’ understandings, experiences, and perceptions of frailty and frailty screening are weak, frailty has become a state priority in South Australia (e.g., SA Health 2014, Frailty Expert Working Group, Transforming Health). In addition, the social construction of health and illness discourses reinforces the fact that experiences and understandings are contextually bound [3, 16]. As such, generating an in-depth understanding of the experiences and perspectives of older adults and healthcare provider groups towards frailty and frailty screening will help in achieving a coordinated frailty and healthy ageing strategy tailored to the South Australian context which might then have relevance for other jurisdictions in Australia and elsewhere. Testing this approach in one context will help illuminate how such evidence can be translated to other jurisdictions.

### Study purpose and objectives

The *“Perspectives of Frailty and Frailty Screening Study of Stakeholder Understandings and Experiences”* is the first phase of a five-year NHMRC funded Centre for Research Excellence (CRE) Grant in Transdisciplinary Frailty Research. The purpose of this research is to understand the experiences and perceptions that diverse stakeholder groups hold about frailty and frailty screening, in order to inform improvements in the prevention, identification, and management of frailty for pre-frail and frail older adults. Specific objectives are to:

1. Identify how key stakeholders (i.e., well, pre-frail, and frail older adults; emergency department (ED) physicians; orthopedic surgeons, and practice nurses and general practitioners (GPs) in urban and rural settings) perceive frailty.
2. Understand stakeholders’ frailty-related experiences
3. Assess stakeholder attitudes towards the concept of frailty screening
4. Assess stakeholders’ perceived feasibility of seven frailty-screening tools validated for use with community-dwelling older people.
5. Identify opportunities for stakeholder-driven and evidence-informed implementation and knowledge translation decisions related to frailty prevention, identification, care, and management in the primary care sector.

## Methods/Design

We will use an exploratory qualitative design combining focus group interviews, face-to-face individual and telephone interviews with stakeholders (e.g., well, pre-frail, and frail older adults, general practitioners, practice nurses, emergency department physicians, orthopedic surgeons) to understand perceptions of frailty and frailty screening within and between these diverse groups. Arts-based data collection methods incorporating visual elicitation (e.g., drawing the meaning of frailty) will also be used with consumer groups to augment qualitative methods. We will iteratively collect and analyze data over a six-month period across urban and rural regions in South Australia (e.g., Adelaide, Southern Fleurieu Peninsula).

### Theoretical Framing

**Fig. 1:**
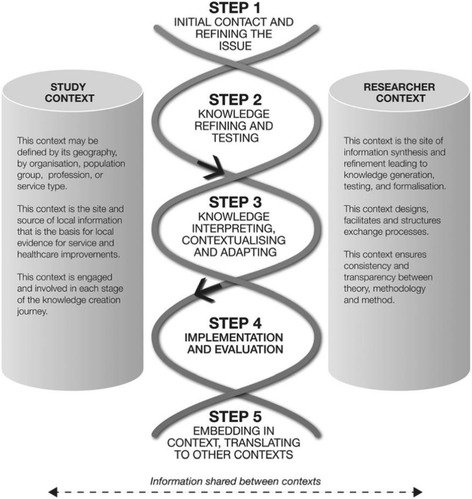
The co-KT Framework [18]. Permission to reuse this figure made available by the Creative Commons License

The Transdisciplinary CRE consists of four research directions, including establishing a new economic model for frailty, identifying cost effective frailty interventions, determining and mapping the prevalence of frailty in South Australia, and implementing a frailty-screening tool in General Practice [17]. Each research direction is underpinned by the co-KT framework [18]– an integrated knowledge translation approach (iKT) that stipulates closer partnerships with stakeholders from the onset of research [18], Fig. 1. Knowledge generated through the proposed study will signify the beginning of a collaborative knowledge production model, wherein emerging study findings concurrently help refine the research problem and inform future study designs. Using this co-production model, research findings and their implications are also shared with stakeholders as they emerge, rather than at the end of the research, as is conventional with end-of-grant KT strategies [19]. This co-production approach capitalizes on the ideal position of stakeholders as knowledge conduits who take and share knowledge within their institutions while providing feedback to the CRE.

### Setting

The study is being conducted in the South Australian capital of Adelaide (population 1.3 million) and surrounding rural areas (Southern Fleurieu Peninsula, Mount Gambier) using remote access technology (e.g., Zoom). These areas differ by population density, remoteness, demography, and health service delivery, thereby providing valuable comparative data. While South Australia’s population is predominantly urban, health services available to older people are unevenly distributed [20]. By 2021, significant changes to South Australia’s age structure will occur– over 47% of the population will be aged 64 and older. These unique population demographics and corresponding scarcity of research into frailty in Australia will allow us to generate useful data to inform stakeholder-driven health services modifications.

### Sample and Inclusion Criteria

Healthcare provider groups were identified based on the frequency of care provided to older adults along the frailty spectrum, and based on the clinical domains represented within the CRE. Other healthcare provider groups will be targeted if needed based on our emerging qualitative data. We will use purposive, convenience sampling as follows:

1. **Well Consumer groups**: We will sample 10–32 adults over aged 50 (2–4 focus groups; sample size driven by data analysis).
2. **Pre-frail and Frail Consumer Groups:** We will sample 10–32 adults over aged 65 (2–4 focus groups; sample size driven by data analysis) from residential care and community settings. Older adults with cognitive capacity to consent and who identify as being pre frail or frail will be included in the study.
3. **Emergency Department Physicians:** We will sample 8–16 emergency department physicians (registrars and consultants) from two emergency departments, one in central Adelaide and one in North Adelaide.
4. **Orthopedic Surgeons:** We will sample 15–24 orthopedic surgeons from Adelaide and broader South Australia. Junior Registrars (i.e., those who have not yet completed their examination requirements), Junior Consultants (i.e., those who have completed their examinations requirements < 10 years ago), and Senior Consultants (i.e., those who have been practicing as a consultant for ≥10 years) will be sampled and data collected in each category.
5. **Practice Nurses ** We will sample 10–32 practice nurses over 2–4 focus groups. Approximately one-third of practice nurse participants will be sampled from non-metropolitan South Australia, with the remainder drawn from metropolitan Adelaide.
6. **General Practitioners** We will sample 15–32 general practitioners over 2–4 focus groups. Approximately one-third of these will be drawn from non-metropolitan South Australia, with the remainder drawn from metropolitan Adelaide. The metropolitan group will be sub-divided by age, with separate focus groups conducted for those aged over 40 and those 40 years and under.

A hallmark characteristic of rigorous qualitative research is that data collection and data analysis occur iteratively, which guides decisions about sample size. As such, it is conventional to produce a sample size “estimate” or range of estimated participants needed to establish a valid result. We have estimated the following sample sizes for our various focus groups and/or individual interviews (Table 1).

**Table 1:** Sample size estimates

Sample	Data Collection & Sample	Site
Well Consumers	2–4 Focus Groups (approximately 5–8 participants / group)	Large metropolitan aged care association; University of the 3rd Age
Pre-Frail and Frail Consumer	2–4 Focus Groups (approximately 5–8 participants / group)	Large metropolitan aged care association and facility
Emergency Department Physicians	Individual Interviews (8–16 participants)	Two metropolitan acute care hospitals (emergency departments)
Orthopedic Surgeons	Individual Interviews (15–24 participants)	Urban, Rural South Australia
Practice Nurses	2–4 Focus Groups (approximately 5–8 participants / group)	Urban (Adelaide Primary Health Network [PHN]) Rural (Country PHN)
General Practitioners	3–4 Focus Groups (approximately 5–8 participants / group)	Urban, Rural South Australia

### Recruitment strategies

#### Older Adults

Older adults will be recruited using site staff or coordinators at two aged care providers in urban and rural Adelaide, and from a continued learning University for adults over 50 in Adelaide. An information poster and information sheet about the research study will be provided to coordinators and administrators at each recruitment site, who will identify potential participants for the focus group study. Prospective participants will be informed about the study from coordinating staff or can consent to be contacted by a member of the research team to learn more about the research study. Degree of frailty status will be confirmed by self-assessment (i.e., whether or not a person associates with being pre-frail or frail) and by the simple Frail questionnaire-screening tool obtained during demographic data collection [21]. For residents in aged care facilities, degree of frailty status will be confirmed using the FRAIL-NH tool [22].

*Orthopedic Surgeons:* Using the established network of orthopedic surgeon and co-author MJC, we will purposively identify 15–24 orthopedic surgeons at various stages of their career trajectories. Individuals will be contacted by telephone to discuss the research study and will be provided with a comprehensive information sheet about the study. Consenting participants will complete a 30–60 min telephone or in-person interview at the initial point of contact or at an established time point in the future. Participants can consent to be contacted in the future for additional data collection or to clarify study findings.

*Emergency Department [ED] Physicians:* Site directors at two Adelaide Emergency Departments will distribute an information sheet about the research study to ED staff. A CRE physician-research affiliate will then approach individuals at the workplace and in his professional network regarding to study to assist in recruiting 8–16 participants. To facilitate recruitment, we will offer to conduct interviews on-site with an option of telephone interviews.

*Practice Nurses:* The Adelaide and Country SA Primary Health Networks will distribute an expression of interest request for participation in the study to their distribution lists via standard communication channels (e.g. newsletters). Practice nurses expressing interest will be approached to invite additional eligible colleagues via a ‘snowball’ recruitment methodology. Consenting participants will attend either an in-person or virtual focus group depending on their location and preference.

*General Practitioners [GPs]:* Investigators JB or RA will approach metropolitan and rural GPs using a convenience sampling approach (i.e. using informal networks of the research team). We will approach GPs who express interest in the study to invite additional eligible GPs via a ‘snowball’ recruitment methodology. To facilitate recruitment, we will offer either in-person or virtual focus groups to consenting participants depending on their location and preference.

### Data collection

We will conduct a series of focus groups, individual and telephone interviews with five stakeholder groups (i.e., well older adults over 50 years of age; pre-frail and frail older adults; ED physicians, GPs, practice nurses, orthopedic surgeons) in South Australia. Focus groups will be conducted with approximately 5–8 individuals to encourage diversity in the information shared in a comfortable group size for participants. The focus groups will last for approximately one to one and a half hours; telephone and in-person interviews will last 30–60 min each.

Researchers with experience in qualitative interviewing will conduct the focus groups, and a minimum of two researchers will be present at each focus group. The purpose of having a second researcher present is to document interactions between group members, which is an important source of focus group data. Focus groups can generate interactive data to reveal consensus and divergent perceptions, especially useful for developing knowledge around issues that are not as well understood. Participant discussion in focus groups can also illuminate natural language used to discuss frailty [23].

Participants will be notified and required to consent to verbatim audio-recording of the focus groups to ensure accurate transcription and interpretation of study findings. When possible, a visual artist will be present during the focus groups to create visual depictions of data; this approach can aid in data analysis and can be a useful way for participants to visualize and make meaning from complex discussions. Other methods of collecting data, such as arts-based and visual elicitation methods, will also be used to better understand participants’ perceptions of frailty and healthy ageing with the consumer groups. Drawing about frailty and its impact will enable insights into representations and health-illness narratives not possible through verbal means alone [24], thereby augmenting and enhancing the focus group research. M.A has experience in arts-based research methods and will lead all aspects incorporating arts-based research approaches.

Research questions will center on the meaning of frailty; the trajectory of frailty, including how it develops and progresses; whether frailty can be detected, prevented, delayed or reversed; the perceived role of health professionals in preventing and treating frailty; perspectives on the concept of frailty screening; impressions of where, how, and by whom frailty screening should occur (e.g., practice nurses during home visits, general practitioners, older adults); and impressions of seven validated frailty screening tools (i.e., Edmonton Frail Scale, Groningen Frailty Index, PRISMA-7, Gait Speed, Timed Up and Go, The Frail Questionnaire, The Kihon Checklist). All interviews will commence with general questions, such as “what does the word frailty mean to you”, and proceed with more specific prompts and questions to clarify meaning and derive more specific understandings.

### Data management and analysis

We will use transcript-based analysis, which enables the greatest degree of rigor while analyzing focus group data [25]. Audio recordings will be repeatedly listened to and transcribed verbatim using a standardized layout and agreed upon notation. We will allocate unique identifiers to each participant and corresponding individual or focus group interview (e.g., the fourth participant in focus group two will be identified as FG2–04). Unique identifiers will be applied to all documents, including field notes, drawings, analytic memos, transcripts, and saved audio files. Data will be managed using NVivo software.

We will use an inductive approach to data analysis guided by qualitative description [26]. We generally will treat the group as the unit of analysis and will code transcripts by lines, and supplement these with field notes and corresponding analytic memos. We will keep reflective analytic notes during the coding process as an audit trail to identify and concurrently justify early themes through analysis [27]. We will use this process during the first few interviews until we establish a flexible coding framework, which we will then apply to subsequent interviews and modify iteratively. During this process, we will pay attention to individuals who did not contribute to the focus group discussion to avoid an artificial interpretation of consensus within the focus group [25]. We will construct a coding matrix to ascertain extent of agreement or alignment and disagreement or misalignments between participants’ perspectives [25]. We will share the coding framework and early themes during collaborative analysis meetings with co-investigators who have independently analyzed data. We will discuss differences and similarities in concepts, sub-themes, and themes, which may result in a new conceptual framework following interrogation of the analytic process and associated outcomes.

Two research team members will inductively analyze visually elicited data from the older adult subgroups to generate a coding framework, drawing from principles of qualitative content analysis. Building from the inductive qualitative approaches used by Luthy and colleagues [28] and others, coding will cover the following dimensions (Table 2):

**Table 2:** Visual coding framework

Dimension	Description	Example
Constituent Elements	All components included in the drawings including perceptions of limitations, changes, difficulties	Anatomical structures Walking aides Housing
Configuration	How constituent elements are positioned in relation to one another	An individual within a house, versus an individual with no external structures represented
Size	In millimeters	Size of total drawing and associated constituent elements

Two researchers will independently code the drawings using a constant comparison method, meaning the inductively generated coding framework will be applied to all drawings while concurrently comparing responses with those previously generated [28]. Once coding is complete, the researchers will confer to explore any discrepancies between their understandings of the visual representations and to achieve consensus. Findings from the visual and text-based analysis will be compared to explore discordances and to maximize trustworthiness and theoretical sensitivity.

## Discussion

Very few studies, particularly in the Australian setting, have explored stakeholder perspectives of frailty and frailty screening [2, 15]. No research has systematically explored the perspectives of various healthcare provider and consumer groups in relation to the experiences, prevention, trajectory, and treatment of frailty. It is important that the perspectives of older people and healthcare providers involved in the care of well, pre-frail and frail older adults is understood, so that strategies can be devised that align with and respect these perspectives. Further, early stakeholder engagement can increase the relevance and impact of research being produced by deliberately aligning research activities with stakeholder experiences and priorities, thereby facilitating KT [17].

Generating a deeper understanding of how well, pre-frail, and frail older adults understand, represent, and experience frailty is an imperative yet overlooked component in current approaches to frailty identification and management. Integrating visual methods with qualitative approaches can augment understanding by eliciting summative representations– simplified statements about the reality of experience that often extend non-visual description [28]. These visual elicitation techniques can provide insights into the often-masked experiential aspects of frailty, a necessary complement to a frailty discourse which has been criticized for emphasizing the physiological components and boundaries of frailty over the emotive and experiential aspects [29]. Yet, understanding the emotional aspects of frailty, alongside its physiological, social and contextual orientations is critical to negating the negative effects of the frailty-identity and optimizing strategies for healthy ageing.

Visual data can further facilitate research engagement and communication [30]. Using the arts in data collection and knowledge translation contests the perennial challenge of representation faced by researchers and participants alike– language, like the selection of research method, is both constraining and liberating, shaping what is expressed and known about phenomena [24]. Participant drawings enable access and insights into important aspects of illness representation [28], which can in turn be used to foster deeper stakeholder understandings across health care provider, researchers, policy, and public spheres. Such insights will provide a needed dimension to the frailty discourse, which has not yet accounted for the multitude of experiential and perceptual understandings of involved stakeholder groups.

The *Perspectives of Frailty and Frailty Screening* study positions these components in dialogical tension, and proposes to use the perceptual understandings of multiple stakeholders as the foundation for a collaborative KT approach and to guide a consolidated frailty strategy in Australia. The findings and the integrated approach to stakeholder engagement is likely to have merit to other jurisdictions – the collaborative approach can function as a transferable model for other states and regions seeking context-sensitive strategies to identifying, preventing, and managing risk and conditions across the health-illness spectrum. Future CRE research building upon this protocol will involve testing the nature and extent of the effectiveness of this approach.

Aligned with the co-KT framework underpinning our research, we conceptualize KT as an iterative, interactive, and collaborative process [17]. The current proposal, which is embedded within the first two steps of the co-KT framework (i.e., refine the issue; knowledge refinement and testing) and to a lesser degree, step three: knowledge interpretation, contextualization, and adaptation, will directly inform subsequent initiatives within the CRE. Specifically, these qualitative findings will help inform a context-driven implementation and evaluation of a frailty-screening tool; drive awareness, knowledge, and skills development strategies across stakeholder groups; and guide future efforts to embed emerging knowledge about frailty and its management across diverse contexts.

## Abbreviations

co-KT: Collaborative Knowledge Translation
CRE: Centre for Research Excellence
ED: Emergency Department
GP: General Practitioner
iKT: Integrated Knowledge Translation
KT: Knowledge Translation
PHN: Primary Health Network

## References

[1] Choi J, Ahn A, Kim S, Won CW. Global Prevalence of Physical Frailty by Fried's Criteria in Community-Dwelling Elderly With National Population-Based Surveys. J Am Med Dir Assoc 2015; doi: http://dx.doi.org/10.1016/j.jamda.2015.02.004 10.1016/j.jamda.2015.02.004 25783624

[2] Blyth FM, Rochat S, Cumming RG, Creasey H, Handelsman DJ, Le Couteur DG, Naganathan V, Sambrook PN, Seibel MJ, Waite LM (2008). Pain, frailty and comorbidity on older men: the CHAMP study. Pain.

[3] South Australia Health. Prosperity through longevity: South Australia’s aging plan. Our vision 2014–2019/Office for the Aging. 2013. ISBN 9781742436050 (paperback) October 2013.

[4] Puts MTE, Shekary N, Widdershoven G, Heldens J, Deeg DJH. The meaning of frailty according to Dutch older frail and non-frail persons. J Aging Stud 2009; doi: http://dx.doi.org/10.1016/j.jaging.2008.03.002

[5] Warmoth K, Lang IA, Phoenix C, Abraham C, Andrew MK, Hubbard RE, Tarrant M (2015). ‘Thinking you're old and frail’: a qualitative study of frailty in older adults. Ageing Soc.

[6] Rowe J. Beyond the bedside: factors influencing the prevalence and management of frailty. In: White Book on Frailty. IAGG GARN Global Aging Research Network. 2016. http://www.garn-network.org/documents/WHITEBOOKONFRAILTY-USVERSION.pdf. Accessed 10 Oct 2016.

[7] Cha, HB. IAGG mission for frailty of older persons. In: White Book on Frailty. IAGG GARN Global Aging Research Network. 2016. http://www.garn-network.org/documents/WHITEBOOKONFRAILTY-USVERSION.pdf. Accessed 10 Oct 2016.

[8] OECD. Social expenditure statistics. 2015. http://www.oecd-ilibrary.org/social-issues-migration-health/data/oecd-social-expenditure-statistics_socx-data-en

[9] Apóstolo J, Cooke R, Bobrowicz-Campos E, Santana S, Marcucci M, Cano A (2016). Effectiveness of the interventions in preventing the progression of pre-frailty and frailty in older adults: a systematic review protocol. JBI.

[10] European Commission (2013). European Innovation Partnership on Active and Healthy Ageing. Prevention and early diagnosis of frailty and functional decline, both physical and cognitive, in older people. Action Group 3. Retrieved from https://ec.europa.eu/research/innovation-union/pdf/active-healthy-ageing/gp_a3.pdf

[11] The Orlando Frailty Conference Group (OFCG) (2013). Raising awareness on the urgent need to implement frailty into clinical practice. J Frailty Aging.

[12] Vellas B. White book on frailty. IAGG GARN Global Aging Research Network. 2016. http://www.garn-network.org/documents/WHITEBOOKONFRAILTY-USVERSION.pdf. Accessed 10 October 2016.

[13] Nessighaoui H, Lilamand M, Patel KV, Vellas B, Laroche ML, Dantoine T, Cesari M (2015). Frailty and pain: two related conditions. J Frailty Aging.

[14] Cameron I, Fairhall N, Langron C (2013). A multifactorial interdisciplinary intervention reduces frailty in older people: randomized trial. BMC Med.

[15] Collard RM, Boter H, Schoevers RA, Oude Voshaar RC (2012). Prevalence of frailty in community-dwelling older persons: a systematic review. J Am Geriatr Soc.

[16] Kaufman S (1994). The social construction of frailty: an anthropological perspective. J Aging Stud.

[17] Archibald M, Kitson A, Frewin D, Visvanathan R. Transdiscplinary research in frailty: knowledge translation to inform new models of care. J Frailty Aging. 2017. (online ahead of print). http://dx.doi.org/10.14283/jfa.2017.6. 10.14283/jfa.2017.6 28555704

[18] Kitson A, Powell K, Hoon E, et al. Knowledge translation within a population health study: how do you do it? Implement Sci. 2013. doi:10.1186/1748-5908-8-54. 10.1186/1748-5908-8-54 PMC3674953 23694753

[19] Canadian Institute of Health Research (CIHR). Guide to knowledge translation planning at CIHR: integrated and end-of-grant approaches. 2015. http://www.cihr-irsc.gc.ca/e/45321.html. Accessed 20 Nov 2016.

[20] Giles L, Halbert J, Gray L, Cameron I, Crotty M (2009). The distribution of health services for older people in Australia: where does transition care fit?. Aust Health Rev.

[21] Morley JE, Vellas B, van Kan GA, Anker SD, Bauer JM, Bernabei R, Cesari M, Chumlea WC, Doehner W, Evans J (2013). Frailty consensus: a call to action. J Am Med Dir Assoc.

[22] Theou O, Tan EC, Bell JS, Emery T, Robson L, Morley JE, Rockwood K, Visvanathan R (2016). Frailty Levels in Residential Aged Care Facilities Measured Using the Frailty Index and FRAIL-NH Scale. J Am Geriatr Soc.

[23] Morgan D. Focus groups and social interaction. In: Gubrium J, Holstein J, Marvasti A, McKinney K, editors. The SAGE Handbook of Interview Research (2nd ed.), Edition: 2nd. Thousand Oaks: SAGE; 2012. p. 161–176.

[24] Archibald M, Caine B, Scott S. Intersections of the arts and nursing knowledge. Nursing Inquiry. 2016. [online first] doi:10.1111/nin.12153. 10.1111/nin.12153 27572849

[25] Onwuegbuzie AJ, Dickinson WB, Leech NL, Zoran AG (2009). A qualitative framework for collecting and analyzing data in focus group research. Int J Qual Methods.

[26] Sandelowski M (2006). What ever happened to qualitative description?. Res Nurs Health.

[27] Chandler C, Reynolds J. ACT consortium guidance: Qualitative research protocol template with example tools and SOPs. 2013. Available at http://www.actconsortium.org/data/files/resources/72/ACTc-Guidance.-Qualitative-methods-for-international-health-intervention-research_Dec2013.pdf. Accessed 10 Nov 2016.

[28] Luthy C, Cedraschi C, Pasquina P, Uldry C, Junod Perron N, Janssens JP (2013). Perception of chronic respiratory impairment in patients drawings. J Rehabil Med.

[29] Grenier A (2006). The distinction between being and feeling frail: exploring emotional experiences in health and social care. J Soc Work Pract.

[30] Archibald M, Caine V, Scott SD. The development of a classification schema for arts-based approaches to knowledge translation. Worldviews Evid Based Nurs. 2014;11(5):316–24. doi:10.1111/wvn.12053. 10.1111/wvn.12053 25132050

